# Supplementary material for: Drought drives rapid shifts in tropical rainforest soil biogeochemistry and greenhouse gas emissions
Source: Nat Commun. 2018 Apr 9;9:1348. doi: 10.1038/s41467-018-03352-3 (PMC5890268; doi:10.1038/s41467-018-03352-3)
Supplement: Supplementary file 1 — Supplementary Information [file 41467_2018_3352_MOESM1_ESM.pdf]

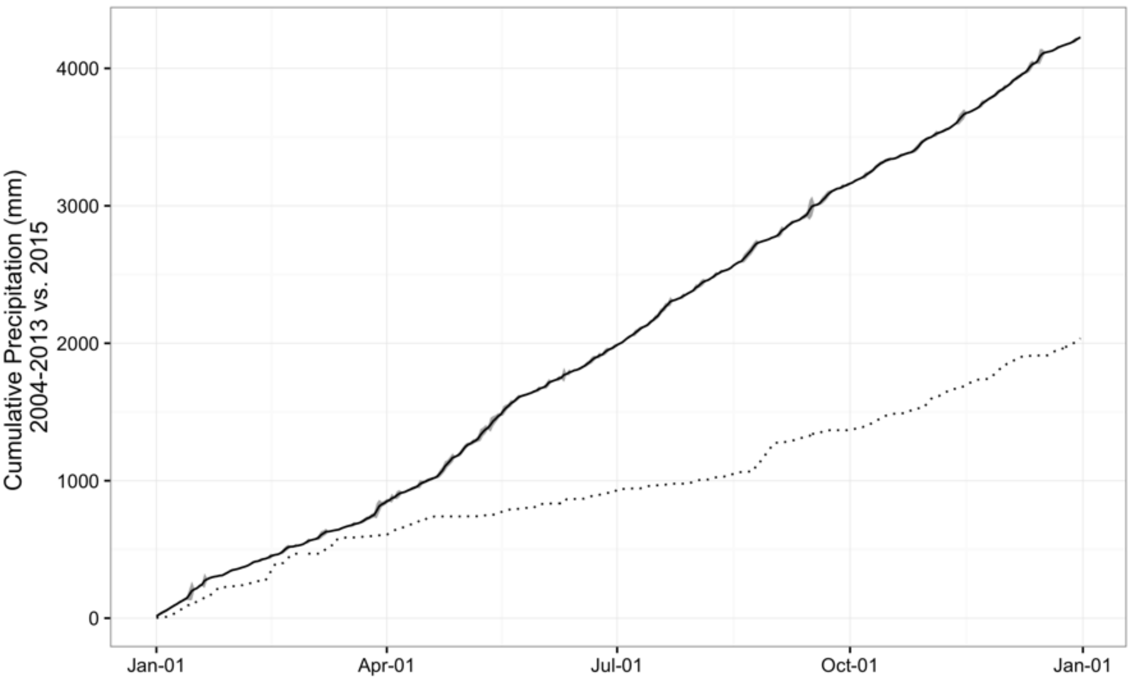

Supplementary Figure 1. Precipitation accumulation graph, 2015 (dashed) in comparison to recent average (solid, 2004-13).

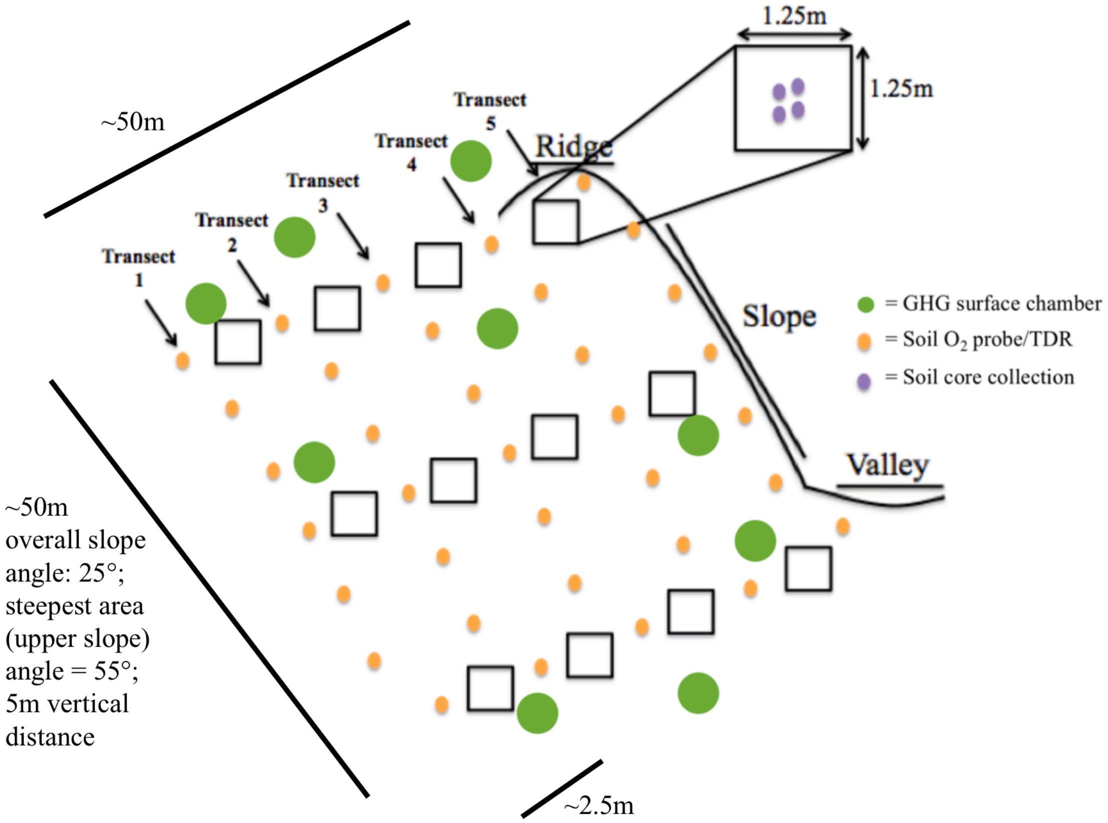

Supplementary Figure 2. Soil moisture and O_2 sensor locations across a topographic gradient in LEF. The sampling array has five transects associated with topographic locations: ridge, upper slope, mid slope, low slope, and valley transects (transects go left to right in the schematic here). Each transect has 7 site locations (5 transects * 7 locations/transect = 35 observations). Each site location is, more precisely, the location of two sensors, one to record soil moisture/temperature and a second to record soil O_2. Data is recorded hourly. Nine automated GHG flux chambers were placed in the array (three each in ridge, slope and valley locations). Soil cores were taken from 0-15 on ridge, slope and valley locations (four replicates per topographic locations) at several time points over the study period.

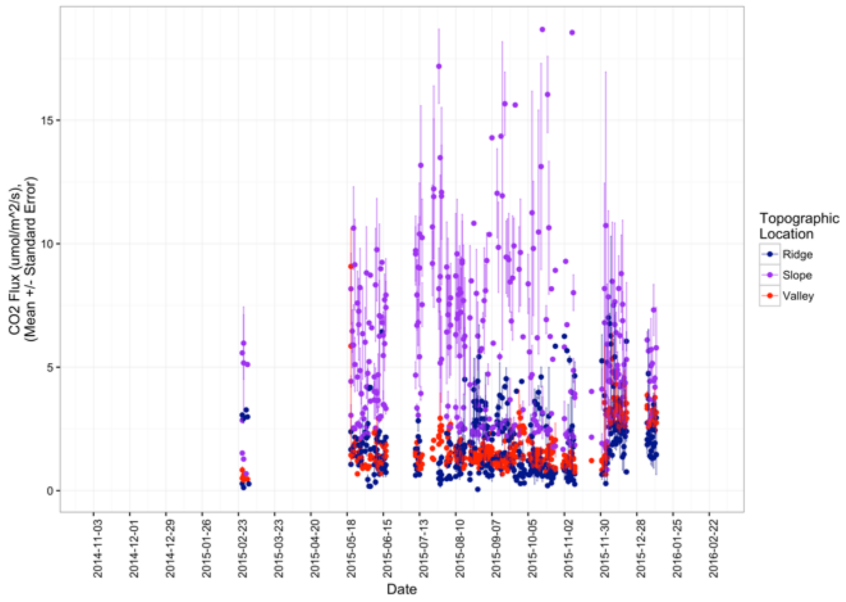

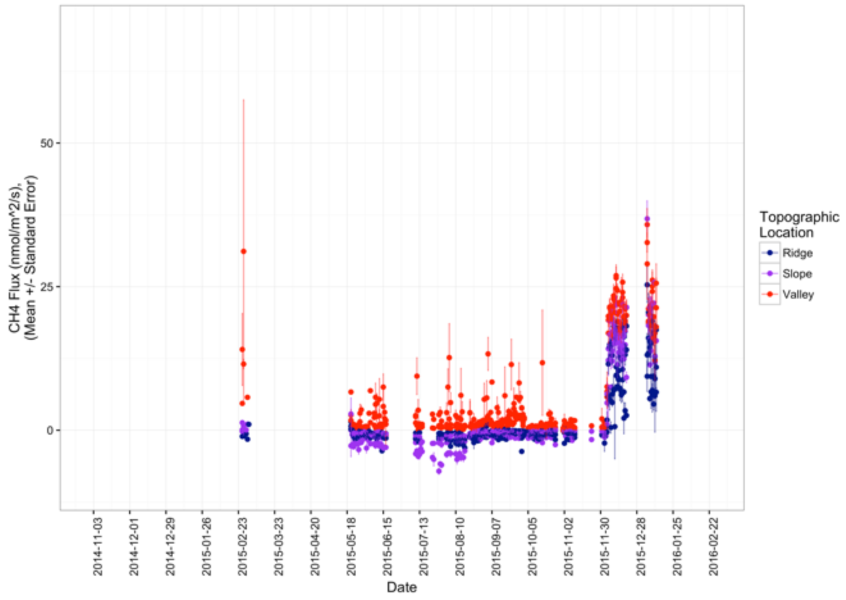

Supplementary Figure 3. Fluxes over time of carbon dioxide (top) and methane (bottom), divided by topographic location (ridge, slope or valley chambers). Data are the daily mean (+/- 1 standard error) of each of the nine deployed chambers (three ridge, three slope, three valley).

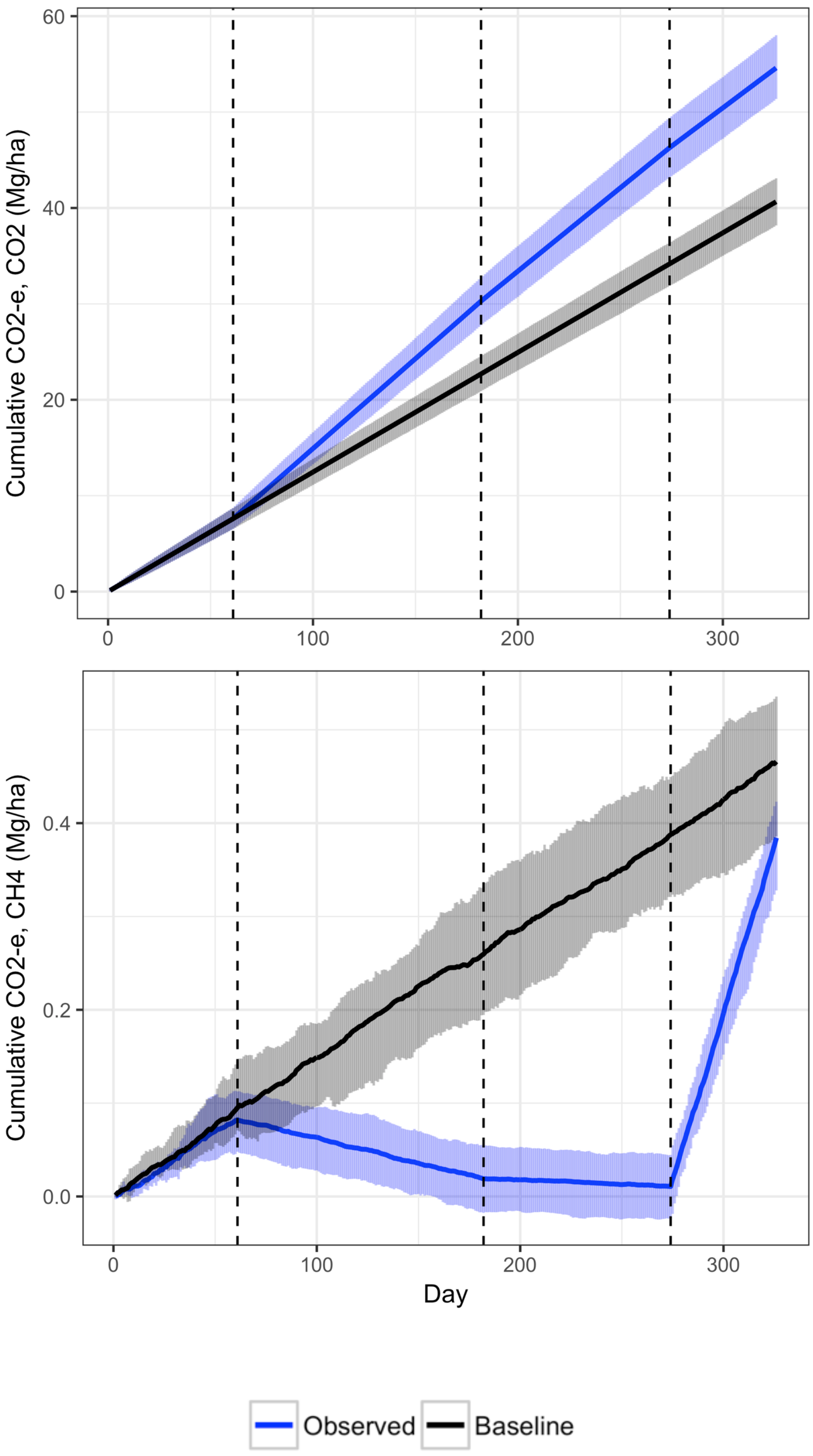

Supplementary Figure 4. Cumulative emissions in Mg ha^{-1} of CO_2 equivalents (CO_2-e) emitted via CO_2 (top) and CH_4 (bottom) over the course of the 326-day study period. Two simulations

were run to estimate the cumulative emissions: a baseline simulation (black line and error bars), in which the pre-drought GHG emissions were presumed to continue, and an observed drought simulation (blue line and error bars), in which cumulative emissions respond to the observed drought periods (Materials and Methods). The lines represent the mean of 5000 simulation runs and the error bars represent the 95% confidence interval. Vertical lines represent the drought periods defined in Figure 2.

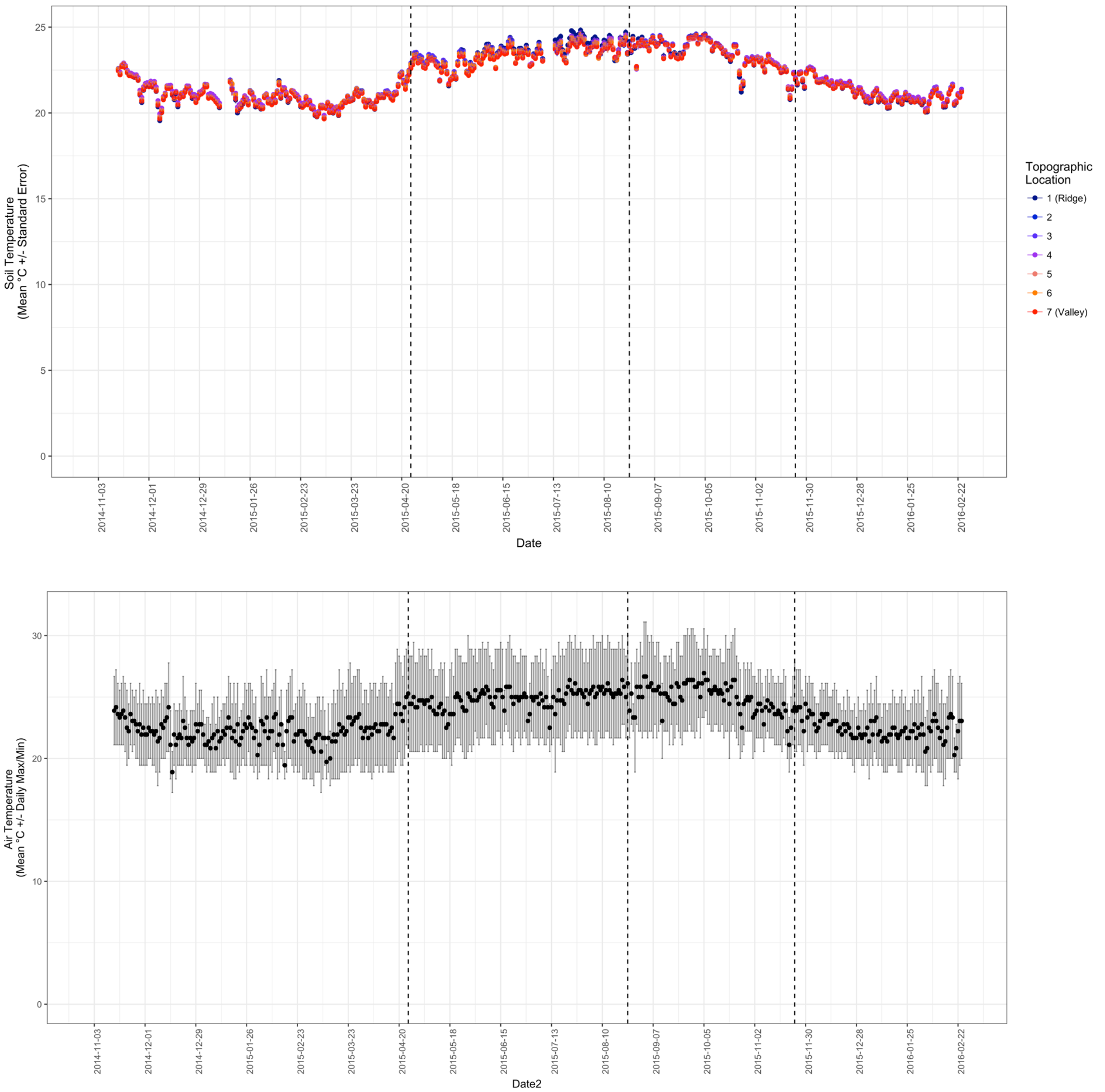

Supplementary Figure 5. Time series of soil temperature (top) and local air temperature (bottom, in degrees Celsius) from Nov. 2014 to Feb. 2016. In the top panel, points are the daily mean for each of the seven topographic locations (ridge to valley); error bars represent the standard error. In the bottom panel, points are the daily mean as reported in the CLIMOD 2 dataset (http://climod2.nrcc.cornell.edu/, accessed September 7, 2017); error bars are the reported maximum and minimum temperature for each day (no standard deviation or error is included in the dataset). The weather station from which air temperature data was taken is station ID 666725, located at 18.3169, -65.8664 degrees, which is 4.96 kilometers from El Verde Research Station.

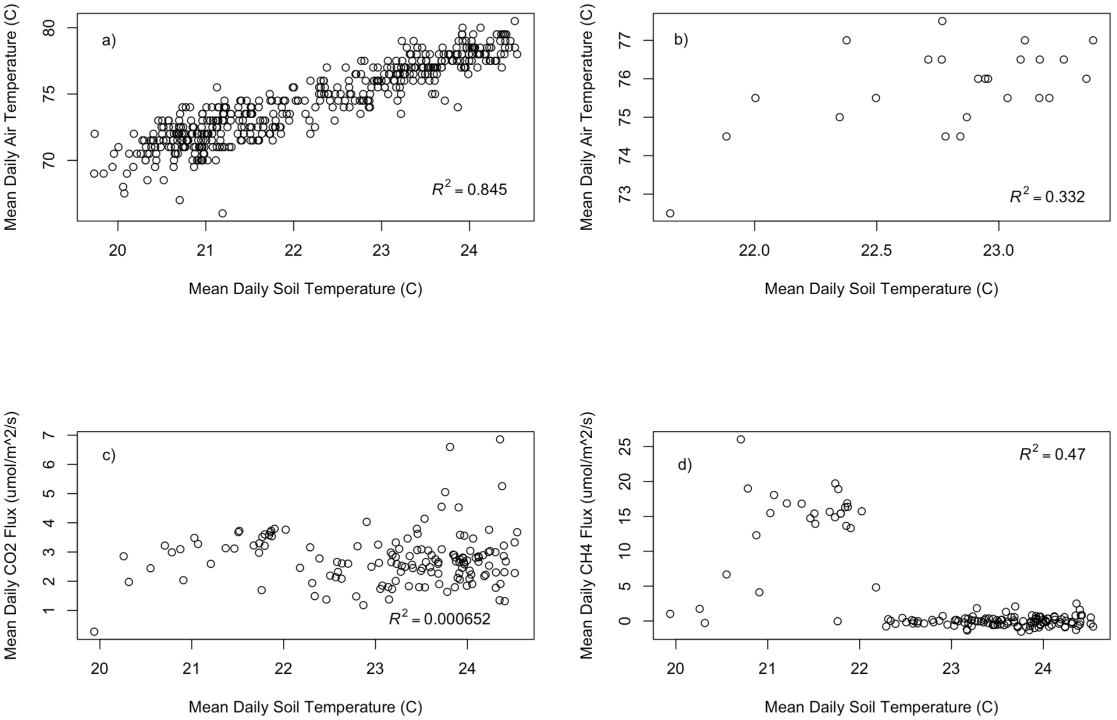

Supplementary Figure 6. Scatterplot of mean daily soil temperature vs mean daily air temperature from (a) all days during the study period and (b) from two days before the drought onset (2015-04-25) to 21 days after the drought onset. Soil temperature data is from the field sensor array while air temperature data is taken as reported in the CLIMOD 2 dataset (http://climod2.nrcc.cornell.edu/, accessed September 7, 2017). In (c) and (d) we show the relationship between the mean daily flux across topographic zones of CO_2 and CH_4 respectively versus the mean soil temperature.

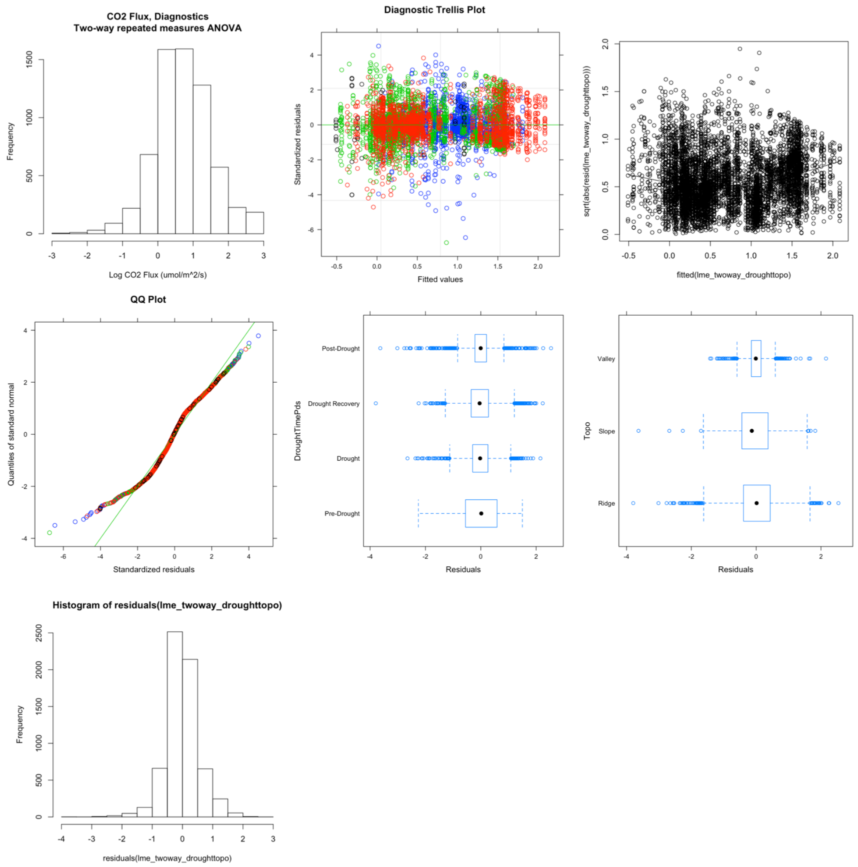

Supplementary Figure 7. Diagnostic plots for a repeated measures two-way ANOVA modeling soil CO_2 emissions. The model estimated CO_2 fluxes (log transformed) using drought time period and topographic location as categorical predictor variables and used measurement date to incorporate repeated measurements. For boxplot diagnostic plots, boxplot whiskers are 1.5 times the interquartile range of the data (defined using base R (R v. 3.2.2)).

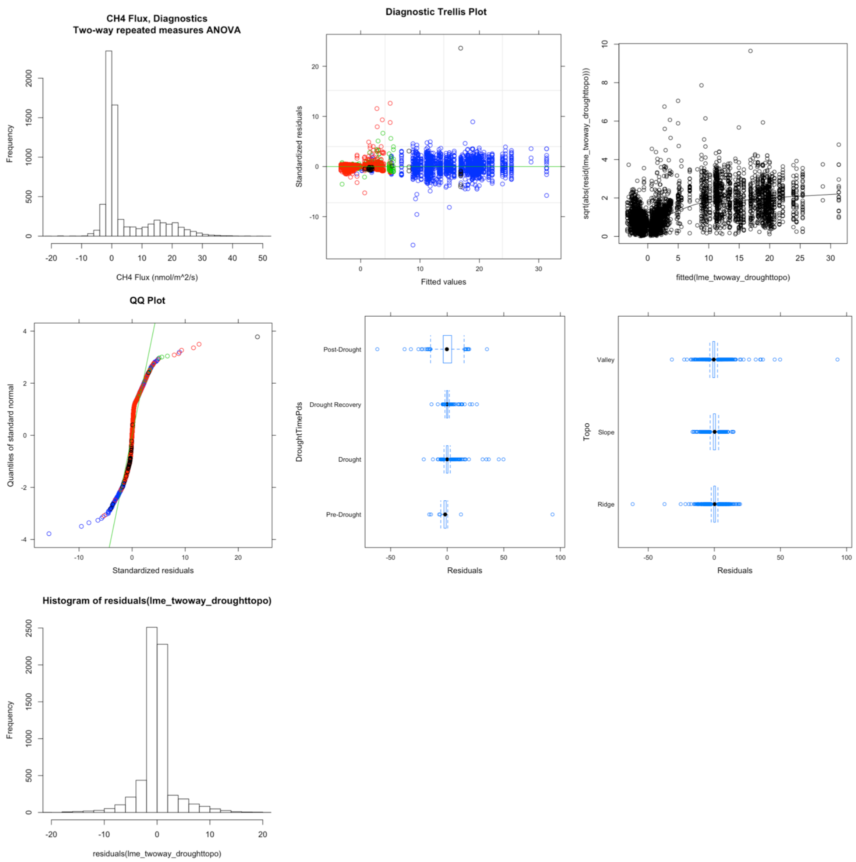

Supplementary Figure 8. Diagnostic plots for a repeated measures two-way ANOVA modeling soil CH_4 emissions. The model estimated CH_4 fluxes using drought time period and topographic location as categorical predictor variables and used measurement date to incorporate repeated measurements. For boxplot diagnostic plots, boxplot whiskers are 1.5 times the interquartile range of the data (defined using base R (R v. 3.2.2)).

Topographic Location	Depth (cm)	Sand (%)**	Silt (%)***	Clay (%)^{NS}
Ridge	0-15	47.13 (0.94)^{a}	29.62 (0.55)^{a}	23.26 (1.48)
Slope	0-15	56.76 (3.00)^{b}	22.92 (1.41)^{b}	20.33 (3.03)
Valley	0-15	60.39 (0.87)^{b}	16.65 (0.93)^{c}	22.96 (0.12)

Supplementary Table 1. Soil texture (mean +/- 1 standard error) across the topographic gradient. Statistical results are reported from a one-way ANOVA in which topographic location predicted the given soil variable; effects are marked as *** when p-value <0.001, ** when p-value <0.01, * when p-value <0.05 and as NS when p-value >= 0.05.

Topographic Location	Depth (cm)	C (%)[NS]	N (%)[NS]	Bulk density (g/cm^3)[NS]
Ridge	0-15	4.83 (0.40)	0.36 (0.035)	0.557 (0.039)
Slope	0-15	3.39 (0.49)	0.27 (0.034)	0.564 (0.059)
Valley	0-15	4.80 (0.65)	0.29 (0.027)	0.755 (0.015)

Supplementary Table 2. Soil properties (mean +/- standard error) across the topographic gradient. Statistical results are reported from a one-way ANOVA in which topographic location predicted the given soil variable; effects are marked as *** when p-value <0.001, ** when p-value <0.01, * when p-value <0.05 and as NS when p-value >= 0.05.

Drought Time Period	Topographic Location	Variable	Mean	SD	SE	CI	N
Pre-Drought	Ridge	CO2 Flux (umol/m^2/s)	1.830	1.468	0.424	0.933	12
Pre-Drought	Slope	CO2 Flux (umol/m^2/s)	3.794	2.921	0.688	1.453	18
Pre-Drought	Valley	CO2 Flux (umol/m^2/s)	0.572	0.172	0.050	0.109	12
Drought	Ridge	CO2 Flux (umol/m^2/s)	1.510	1.046	0.036	0.071	826
Drought	Slope	CO2 Flux (umol/m^2/s)	6.058	4.256	0.141	0.276	913
Drought	Valley	CO2 Flux (umol/m^2/s)	1.506	0.753	0.025	0.050	884
Drought Recovery	Ridge	CO2 Flux (umol/m^2/s)	1.873	1.633	0.059	0.115	779
Drought Recovery	Slope	CO2 Flux (umol/m^2/s)	5.099	4.281	0.204	0.401	440
Drought Recovery	Valley	CO2 Flux (umol/m^2/s)	1.357	0.474	0.017	0.033	807
Post-Drought	Ridge	CO2 Flux (umol/m^2/s)	2.845	2.810	0.079	0.155	1260
Post-Drought	Slope	CO2 Flux (umol/m^2/s)	2.833	2.294	0.065	0.128	1236
Post-Drought	Valley	CO2 Flux (umol/m^2/s)	1.822	1.432	0.038	0.075	1394
Pre-Drought	Ridge	CH4 Flux (nmol/m^2/s)	-0.636	0.758	0.240	0.542	10
Pre-Drought	Slope	CH4 Flux (nmol/m^2/s)	0.149	0.691	0.168	0.355	17
Pre-Drought	Valley	CH4 Flux (nmol/m^2/s)	17.429	29.606	8.546	18.810	12
Drought	Ridge	CH4 Flux (nmol/m^2/s)	-1.121	1.002	0.035	0.069	814
Drought	Slope	CH4 Flux (nmol/m^2/s)	-1.933	1.726	0.057	0.112	910
Drought	Valley	CH4 Flux (nmol/m^2/s)	1.677	4.089	0.138	0.270	884
Drought Recovery	Ridge	CH4 Flux (nmol/m^2/s)	-0.665	0.661	0.024	0.048	745
Drought Recovery	Slope	CH4 Flux (nmol/m^2/s)	-0.567	0.530	0.027	0.052	398
Drought Recovery	Valley	CH4 Flux (nmol/m^2/s)	1.491	2.562	0.090	0.177	807
Post-Drought	Ridge	CH4 Flux (nmol/m^2/s)	5.095	7.733	0.224	0.439	1194
Post-Drought	Slope	CH4 Flux (nmol/m^2/s)	5.457	9.576	0.286	0.562	1118
Post-Drought	Valley	CH4 Flux (nmol/m^2/s)	15.628	37.399	1.005	1.971	1385

Supplementary Table 3. CO_2 and CH_4 flux variable values across topographic zones (as displayed in Figure 3). For each variable, we report the sample size (N), mean, standard deviation (SD), standard error (SE) and 95% confidence interval width (CI).

Drought Time Period	Topographic Location	Variable	Mean	SD	SE	CI	N
Drought	Ridge	Fe(II) (mg-Fe/g)	0.053	0.019	0.008	0.020	6
Drought	Slope	Fe(II) (mg-Fe/g)	0.057	0.087	0.025	0.055	12
Drought	Valley	Fe(II) (mg-Fe/g)	2.823	3.306	1.349	3.469	6
Pre-Drought	Ridge	Fe(II) (mg-Fe/g)	0.053	0.023	0.009	0.024	6
Pre-Drought	Slope	Fe(II) (mg-Fe/g)	0.048	0.020	0.008	0.021	6
Pre-Drought	Valley	Fe(II) (mg-Fe/g)	14.936	3.744	1.529	3.929	6
Drought	Ridge	Fe(III) (mg-Fe/g)	0.995	0.259	0.106	0.272	6
Drought	Slope	Fe(III) (mg-Fe/g)	1.028	0.981	0.283	0.623	12
Drought	Valley	Fe(III) (mg-Fe/g)	3.368	0.781	0.319	0.819	6
Pre-Drought	Ridge	Fe(III) (mg-Fe/g)	0.961	0.206	0.084	0.216	6
Pre-Drought	Slope	Fe(III) (mg-Fe/g)	1.045	0.449	0.183	0.472	6
Pre-Drought	Valley	Fe(III) (mg-Fe/g)	1.328	0.716	0.292	0.751	6
Drought	Ridge	pH	4.522	0.134	0.055	0.141	6
Drought	Slope	pH	4.899	0.208	0.060	0.132	12
Drought	Valley	pH	5.025	0.177	0.072	0.186	6
Pre-Drought	Ridge	pH	4.537	0.214	0.087	0.224	6
Pre-Drought	Slope	pH	4.912	0.137	0.056	0.144	6
Pre-Drought	Valley	pH	6.123	0.188	0.077	0.197	6
Drought	Ridge	Inorganic P (ug/g)	37.614	6.197	2.530	6.504	6
Drought	Slope	Inorganic P (ug/g)	32.486	13.391	3.866	8.509	12
Drought	Valley	Inorganic P (ug/g)	75.360	24.463	9.987	25.672	6
Pre-Drought	Ridge	Inorganic P (ug/g)	60.429	11.114	4.537	11.663	6
Pre-Drought	Slope	Inorganic P (ug/g)	85.564	13.157	5.371	13.807	6
Pre-Drought	Valley	Inorganic P (ug/g)	189.469	47.373	19.340	49.715	6
Drought	Ridge	Organic P (ug/g)	162.003	41.013	16.744	43.041	6
Drought	Slope	Organic P (ug/g)	149.883	40.843	11.790	25.950	12
Drought	Valley	Organic P (ug/g)	174.695	25.374	10.359	26.628	6
Pre-Drought	Ridge	Organic P (ug/g)	71.797	13.434	5.485	14.099	6
Pre-Drought	Slope	Organic P (ug/g)	95.453	13.414	5.476	14.077	6
Pre-Drought	Valley	Organic P (ug/g)	43.619	17.248	8.624	27.445	4

Supplementary Table 4. Soil biogeochemical variable values before and during drought, including inorganic P, organic P, Fe(II), Fe(III) and pH (as displayed in Figure 4). For each variable, we report the sample size (N), mean, standard deviation (SD), standard error (SE) and 95% confidence interval width (CI).
