# Supplementary material for: Drought drives rapid shifts in tropical rainforest soil biogeochemistry and greenhouse gas emissions
Source: Nat Commun. 2018 Apr 9;9:1348. doi: 10.1038/s41467-018-03352-3 (PMC5890268; doi:10.1038/s41467-018-03352-3)
Supplement: Supplementary file 3 — Description of Additional Supplementary Files [file 41467_2018_3352_MOESM3_ESM.pdf]

Description of Additional Supplementary Files

File Name: Supplementary Data 1
Description: Details of statistical tests performed including sample sizes, degrees of freedom, initial test results and posthoc test results.

File Name: Supplementary Data 2
Description: CO_2 and CH_4 flux variable values during each drought period for each chamber (as displayed in Figure 3 and as summarized in Supplementary Table 3 by topographic zone). For each variable, we report the sample size (N), mean, standard deviation (SD), standard error (SE) and 95% confidence interval width (CI). All data is reported after data cleaning.
